# Male condom and dual protection use by adolescent men in Brazil

**DOI:** 10.11606/s1518-8787.2021055003298

**Published:** 2021-11-23

**Authors:** Ana Luiza Vilela Borges, Luciane Simões Duarte, Cristiane da Silva Cabral, Alejandra Andrea Roman Lay, Osmara Alves Viana, Elizabeth Fujimori

**Affiliations:** I Universidade de São Paulo Escola de Enfermagem Departamento de Enfermagem em Saúde Coletiva São Paulo SP Brasil Universidade de São Paulo. Escola de Enfermagem. Departamento de Enfermagem em Saúde Coletiva. São Paulo, SP, Brasil; II Secretaria de Estado da Saúde de São Paulo Centro de Vigilância Epidemiológica Divisão de Doenças Crônicas Não Transmissíveis São Paulo SP Brasil Secretaria de Estado da Saúde de São Paulo. Centro de Vigilância Epidemiológica. Divisão de Doenças Crônicas Não Transmissíveis. São Paulo, SP, Brasil; III Universidade de São Paulo Faculdade de Saúde Pública Departamento Saúde, Ciclos de Vida e Sociedade São Paulo SP Brasil Universidade de São Paulo. Faculdade de Saúde Pública. Departamento Saúde, Ciclos de Vida e Sociedade. São Paulo, SP, Brasil; IV Universidad de Tarapacá Facultad de Ciencias de la Salud Arica Chile Universidad de Tarapacá. Facultad de Ciencias de la Salud. Arica, Chile

**Keywords:** Adolescent, Men, Contraception, Contraceptives, Oral, Condoms, Sexual and Reproductive Health

## Abstract

**OBJECTIVE:**

To assess the use of male condoms and dual protection by Brazilian adolescent men, as well as their associated aspects.

**METHODS:**

A database from the Study of Cardiovascular Risks in Adolescents (ERICA) was used for this national cross-sectiotabelnal school-based research. The sample included adolescents of both sexes, aged between 12 and 17 years old, selected through cluster sampling in 2014 (n = 75,060). This study analyzed information from adolescent men who reported having had sexual intercourse (n = 12,215). The dependent variables were the use of male condoms and the use of dual protection (simultaneous use of male condoms and oral hormonal contraceptives) in the last sexual intercourse. Data were analyzed using univariate and multiple logistic regression.

**RESULTS:**

Most adolescents used a male condom in the last sexual intercourse, while the use of double protection was quite low. The use of male condoms, reported by 71% (95%CI 68.7–73.1), was positively associated with age, living with both parents, and having used alcohol in the previous 30 days. The use of double protection, reported by 3.6% (95%CI 2.8–4.5) was positively associated with age and studying in a private school, as well as negatively associated with tobacco use in the previous 30 days.

**CONCLUSIONS:**

The wide difference shown in the proportion of condom or dual protection use in the last sexual intercourse draws attention to the different logics that govern juvenile sexual relations. The low proportion of dual protection use may be a reflection of men’s lack of knowledge about a function that has historically been attributed to women, which is contraception. Thus, one must deconstruct such dichotomy that the sphere of sexuality is of the domain/interest of men, while that of reproduction concerns only women.

## INTRODUCTION

Contraceptive practices among adolescents have been quite heterogeneous among high, middle and low-income countries, but they have in common the fact they are limited to the use of short-term methods, such as the male condom and, to a lesser extent, the oral hormonal contraceptive^1–4^. Knowledge about contraceptive behavior in adolescence is mostly found in studies that consider only women^5^. This occurs for several reasons: in general, population surveys are carried out predominantly with women; vital birth statistics provide data only on women who are mothers but not on fathers; the widely available contraceptive methods are primarily for female use; teenage pregnancy rates are estimated considering in their denominator only women in the adolescent age group. In summary, the main reason why studies consider only women when researching contraceptive behavior in adolescence is that women are still held responsible for reproductive planning and childcare^6,7^ in our society. The small presence of men in the universe of research on family planning reflects the male invisibility in public policies and health programs^7^.

The most used contraceptive method by male adolescents is the male condom^1,3,4^. In addition to preventing pregnancy, the male condom is highly recommended for adolescents to prevent sexually transmitted infections (STIs) and HIV/AIDS, both because of its low cost and because it is widely available. Data from the Health Behavior in School-aged Children (HBSC) study, which involved 42 countries, mainly European, showed that 65% of 15-year-old adolescents of both sexes had used the male condom in their last sexual intercourse^8^. A similar result was registered among North American adolescents (54%)^9^. In Brazil, data from the National Adolescent Health Survey (PeNSE), carried out with adolescents aged 13 to 17 years, showed high levels of condom use in the last sexual intercourse, despite a significant drop in the time interval between two moments of the survey (76% in 2009 and dropped to 66% in 2015)^10^.

The use of dual protection (the use of male condoms associated with another contraceptive method to prevent pregnancy) is little known among adolescent men^3,11^. In Brazil, although there is no specific information about the practice among male adolescents, data from PeNSE 2015 found a prevalence of 29.7% of use of dual protection among adolescents who had started their sexual life^10^.

This study assesses the use of male condoms and dual protection among Brazilian adolescent men, as well as their associated aspects. The study is intended to elucidate gaps in the literature on contraceptive practices among men, including adolescents. Providing evidence-based care to adolescent men is important to address their sexual and reproductive health needs, including STI prevention, contraception and parenthood^12^. Undoubtedly, the practice of safe sex among adolescents remains a major challenge for the area of sexual and reproductive health, given the evidence of an increase in HIV/AIDS and STI rates^8,13^ and of the high adolescent fertility rates in Brazil and Latin America^14^.

## METHODS

This is a national school-based cross-sectional study that used the Study of Cardiovascular Risks in Adolescents (ERICA) database. ERICA interviewed 75,060 adolescents of both sexes who attended schools in Brazilian cities with more than 100,000 inhabitants, to assess the prevalence of cardiovascular risk conditions^15^.

Adolescents were selected in 2014 through cluster sampling, considering school, shift, year, and class of public and private institutions. The sample included adolescents aged between 12 and 17 years, from middle school (7th to 9th grade) and high school (1st to 3rd grade). The sample size calculation procedures and sampling plan are published elsewhere^16^. This study analyzed information regarding adolescent men who reported having had sexual intercourse (n = 12,215), which represents 16.3% of the sample.

Sociodemographic and behavioral data were collected through a *Personnal Digital Assistant* and the questionnaire was self-completed by the adolescents in the classroom, under the supervision of the team^15^.

The dependent variables were the use of male condoms and the use of dual protection in the last sexual intercourse, data obtained through the question “The last time you had sexual intercourse, did you or your partner used it (you can mark more than one option): I have never had sexual intercourse; condom; oral contraceptive pill; morning after pill; other”. Dual protection was understood as the simultaneous use of a male condom (condom) and oral hormonal contraceptive (oral contraceptive pill), i.e., when both options were checked. Emergency contraception (morning after pill) was not considered, as it is not a method of regular use.

Independent variables gathering information on sociodemographic and behavioral characteristics were: age (in years), skin color (white or non-white, which included black, brown, yellow, and indigenous), residence (capital or countryside), region of Brazil (North, Northeast, Southeast, South, or Midwest), cohabitation (neither with mother nor father, only with mother, only with father, or both), school network (public or private), paid work (no or yes), economic group (low, medium or high) and alcohol/tobacco use in the previous 30 days (no, only tobacco, only alcohol, or both). Economic groups were built based on the consumer goods the adolescents had at home, according to procedures described to calculate the Wealth Index in the Demographic and Health Survey^17^, considering consumer goods such as television, radio, washing machine, DVD, fridge, freezer, motorcycle, car and computer. The hiring of a maid working at home was also considered.

Data were described using proportions and 95% confidence intervals (95%CI). Aspects associated with the use of male condoms and dual protection in the last sexual intercourse were first analyzed using univariate logistic regression and, later, multiple, with simultaneous entry of variables into the model. Since this is a complex sample, the sample weights were considered in all analyses. A significance level of 5% was considered. All analyzes were conducted on Stata 15.0.

ERICA was approved by the Research Ethics Committees of the Institute for Studies in Collective Health of the Federal University of Rio de Janeiro (Process no. 45/2008) and of each of the 26 states and the Federal District.

## RESULTS

Among the 12,215 male adolescents aged between 12 and 17 years who reported having had sexual intercourse, most were between 15 and 17 years old (70.5%), and self-classified their skin color as non-white (66.2 %), attended public schools (86.9%), lived in the countryside (56.2%), and did not have any paid work (56.9%). Just over half lived with both parents (51.4%) and reported not having used tobacco or alcohol in the 30 days prior to the interview (52.4%). Almost half lived in the Southeast region (49.4%) and were included in the middle economic group (49.0%) (Table 1).

**Table 1:** Sociodemographic and behavioral characteristics of male adolescents in Brazil. ERICA, Brazil, 2013–2014.

Characteristics		Total (n = 12,215)		Male condom use (n = 8,783)		Use of double protection (n = 469)	
		%	CI95%	%	CI95%	%	CI95%
Age (years)							
	12	5.4	4.5–6.5	3.3	2.5–4.4	0.6	0.0–2.9
	13	9.6	8.4–10.9	8.1	6.8–9.6	1.7	0.1–4.3
	14	14.5	13.3–15.8	15.4	13.4–17.3	6.6	39.7–10.7
	15	22.3	20.7–23.9	22.5	20.7–24.5	25.1	16.5–36.3
	16	24.3	22.9–25.8	25.3	23.5–27.1	27.6	21.4–34.7
	17	23.9	22.6–25.2	25.4	23.6–27.2	38.4	31.3–46.1
School network							
	Public	86.9	82.5–90.3	86.9	82.8–90.2	76.8	61.5–87.3
	Private	13.1	9.6–17.5	13.1	9.8–17.1	23.2	12.7–38.5
Skin color							
	White	33.8	31.7–35.9	33.7	31.3–36.2	43.4	31.0–56.7
	Non-white	66.2	64.1–68.3	66.3	63.8–68.7	56.6	43.3–69.0
Residence							
	Capital	43.8	41.5–46.1	43.3	407–45.8	41.8	31.3–53.2
	Countryside	56.2	53.9–58.5	56.7	54.1–59.3	58.2	468–68.7
Brazilian region							
	North	10.5	9.8–11.2	10.3	9.7–11.2	7.3	4.9–10.5
	Northeast	21.9	20.0–23.9	21.0	19.2–22.9	9.9	6.1–15.7
	Southeast	49.4	46.8–52.0	50.0	47.1–52.8	57.7	46.2–68.5
	South	10.3	9.2–11.5	10.6	9.1–12.3	15.6	10.3–22.9
	Midwest	7.9	7.0–8.9	8.1	6.9–9.4	9.5	5.9–14.8
Cohabitation							
	Neither with mother nor father	7.7	6.3–9.3	7.2	6.1–8.4	4.3	2.6–6.9
	Only with mother	35.2	33.3–37.3	34.8	32.6–37.1	40.2	29.1–52.5
	Only with father	5.7	5.0–6.6	5.3	4.4–6.3	5.5	27.5–10.7
	Both	51.4	48.7–54.0	52.7	50.0–55.4	50.0	38.1–61.8
Did paid work							
	No	56.9	55.1–58.7	56.4	54.0–58.7	47.7	36.1–59.5
	Yes	43.1	41.3–44.9	43.6	41.3–46.0	52.3	40.4–63.9
Economic Group							
	Low	28.8	26.6–31.0	27.6	25.5–29.9	16.9	11.2–24.6
	Middle	49.0	46.8–51.2	49.4	47.0–51.8	56.5	47.5–65.2
	High	22.2	19.9–24.7	23.0	20.4–25.8	26.6	19.2–35.5
Alcohol/tobacco use in the previous 30 days							
	No	52.4	49.7–55.0	51.1	48.4–53.9	42.1	34.1–50.7
	Tobacco only	2.7	2.2–3.3	2.7	2.1–3.3	0.7	0.3–1.4
	Alcohol only	34.5	32.1–36.9	36.2	33.7–38.9	47.0	38.2–56.0
	Both	10.4	9.2–11.8	10.0	8.6–11.5	10.2	5.9–17.0

Most adolescents used a male condom in the last sexual intercourse (71.7%; 95%CI 68.7–73.1). The use of dual protection was low (3.6%; 95%CI 2.8–4.5).

The association of adolescent male characteristics with the use of male condoms in the last sexual intercourse is shown in Table 2. Adolescents’ age was associated with the use of male condoms (ORadj = 1.23; 95%CI 1.16–1.31). Adolescents who lived with both parents were more likely to use male condoms, in contrast to those who did not live with either parent (ORadj = 1.61; 95%CI 1.04–2.49), as well as those who used alcohol in the 30 days prior, when compared to those who did not use either tobacco or alcohol (ORadj = 1.29; 95%CI 1.00–1.65).

**Table 2:** Association between sociodemographic and behavioral characteristics of male adolescents in Brazil and male condom use in the last sexual intercourse. ERICA, Brazil, 2013–2014.

Characteristics		Male condom use			
		Univariate analysis		Multiple analysis	
		OR_Gross_	CI95%	OR_Adj_	CI95%
Age (years)		1.21	1.14–1.29	1.23	1.16–1.31
School network					
	Public	Ref.	-	Ref.	-
	Private	0.99	0.75–1.31	0.85	0.60–1.20
Skin color					
	White	Ref.	-	Ref.	-
	Non-white	1.01	0.84–1.22	1.07	0.86–1.33
Brazilian region					
	North	Ref.	-	Ref.	-
	Northeast	0.90	0.71–1.14	0.83	0.65–1.06
	Southeast	1.07	0.88–1.32	0.95	0.77–1.17
	South	1.14	0.84–1.55	1.02	0.72–1.44
	Midwest	1.12	0.86–1.46	1.01	0.83–1.37
Residence					
	Capital	Ref.	-	Ref.	-
	Countryside	1.07	0.88–1.30	1.00	0.81–1.22
Cohabitation					
	Neither with mother nor father	Ref.	-	Ref.	-
	Only with mother	1.18	0.77–1.80	1.41	0.91–2.19
	Only with father	0.94	0.62–1.44	0.98	0.63–1.55
	Both	1.35	0.85–2.02	1.61	1.04–2.49
Did paid work					
	No	Ref.	-	Ref.	-
	Yes	1.08	0.88–1.32	1.00	0.80–1.23
Economic Group					
	Low	Ref.	-	Ref.	-
	Middle	1.17	0.98–1.41	1.13	0.89–1.43
	High	1.29	1.01–1.67	1.32	0.97–1.77
Tobacco/alcohol use in the previous 30 days					
	No	Ref.	-	Ref.	-
	Tobacco only	1.01	0.66–1.53	1.02	0.64–1.63
	Alcohol only	1.30	1.03–1.64	1.29	1.00–1.65^a^
	Both	0.93	0.71–1.21	0.94	0.71–1.26

OR: odds ratio; ORadj: adjusted odds ratio; Ref.: reference.

a p = 0.049

Table 3 shows the factors associated with the use of dual protection in the last sexual intercourse among adolescent men. The results showed that age was also associated with the use of double protection, as well as studying in a private school. Tobacco/alcohol use in the previous 30 days was inversely associated with the use of dual protection (ORadj = 0.27; 95%CI 0.10–0.69). Although the variables on region of residence, cohabitation and economic group were found to be associated with the use of dual protection in the univariate analysis, they lost their significance in the multiple model.

**Table 3:** Association between sociodemographic and behavioral characteristics of male adolescents in Brazil and the use of double protection in the last sexual intercourse. ERICA, Brazil, 2013–2014.

Characteristics		Use of double protection			
		Univariate analysis		Multiple analysis	
		OR_Gross_	CI95%	OR_Adj_	CI95%
Age (years)		1.50	1.31–1.71	1.44	1.25–1.67
School network					
	Public	Ref.	-	Ref.	-
	Private	2.07	1.18–3.63	1.72	1.04–2.82
Skin color					
	White	Ref.	-	Ref.	-
	Non-white	0.56	0.37–1.11	0.94	0.56–1.61
Brazilian region					
	North	Ref.	-	Ref.	-
	Northeast	0.65	0.39–1.10	0.62	0.36–1.07
	Southeast	1.74	1.07–2.84	1.29	0.80–2.10
	South	2.29	1.44–3.63	1.65	0.97–2.81
	Midwest	1.79	1.01–3.15	1.50	0.84–2.67
Residence					
	Capital	Ref.	-	Ref.	-
	Countryside	1.09	0.70–1.68	1.23	0.71–1.78
Cohabitation					
	Neither with mother nor father	Ref.	-	Ref.	-
	Only with mother	2.09	1.06–4.11	1.49	0.94–6.64
	Only with father	1.75	0.76–4.02	1.95	0.75–5.06
	Both	1.77	0.93–3.63	1.83	0.73–4.56
Did paid work					
	No	Ref.	-	Ref.	-
	Yes	1.46	0.89–2.40	1.27	0.77–2.10
Economic Group					
	Low	Ref.	-	Ref.	-
	Middle	2.02	1.20–3.39	1.53	0.86–2.70
	High	2.09	1.19–3.68	1.47	0.80–2.70
Tobacco/alcohol use in the previous 30 days					
	No	Ref.	-	Ref.	-
	Tobacco only	0.29	0.13–0.66	0.27	0.10–0.69
	Alcohol only	1.73	1.17–2.57	1.31	0.84–2.03
	Both	1.21	0.65–2.28	0.97	0.48–1.98

OR: odds ratio; ORadj: adjusted odds ratio; Ref.: reference.

## DISCUSSION

Results of this study, which analyzed data from ERICA, contribute to the advancement of knowledge about the use of male condoms and dual protection among adolescent men. In our study, the use of these methods increased with age and was influenced by cohabitation with parents, the use of alcohol and/or tobacco, and the school network the teenager attends.

Observing data obtained on the use of male condoms, the results were higher than those verified in PeNSE 2015^10^, which was 66%, and in the 2017 Youth Risk Behavior Survey, which evaluated students aged 14 to 17 years old in the United States and resulted in 61%^18^. On the other hand, the use was lower than that observed in European countries, which obtained results close to 80%^19^. Of the total number of male adolescents selected in this research, 71.7% used a male condom in their last sexual intercourse.

However, the use of dual protection in the last sexual intercourse was low when compared to other studies. In public schools in Cuiabá, Mato Grosso, Brazil, for example, 9% of male high school adolescents reported a combination of contraceptive methods^20^. According to the National Survey of Family Growth, this proportion reached 19% among American adolescent men^3^, but data from the Youth Risk Behavior Survey showed that only 9% used dual protection methods, a proportion that has remained constant since 2013^9^. Several studies analyze the use of dual protection, considering the use of methods other than the male condom, such as the female condom, intrauterine device, injectables and implants, while our study considered only the male condom and the oral hormonal contraceptive, which makes comparing results tricky. In any case, the use of these other methods not considered here among adolescents has been uncommon in the country^10^.

Age was the only aspect associated with both the use of male condoms and dual protection, confirming that older adolescents tend to use contraceptive methods more, which has been previously observed in other studies^2,3,13^. This also shows that younger adolescents can be the most vulnerable to the consequences of not using contraceptive methods, such as unintentional pregnancy, STIs and HIV/AIDS^3,21^. One aspect that may have contributed to these results would be the barriers to obtaining contraceptive methods, either due to difficulty in accessing the health services that offer them, or because they have less financial resources to obtain them from pharmacies, as well as doubts about how to attain and use contraceptive methods^21^.

Whereas dialogue between partners about the consequences of unprotected sexual intercourse, as well as making explicit the desire to use a method, are essential prerequisites for increasing male condom use among adolescent men^22^, younger men may have greater difficulty and inexperience in dealing with these issues. For this reason, it is important to recognize that sexuality education initiatives must be implemented in early adolescence, even before the onset of sexual life, with access to information and supplies so that adolescent men can experience their sexuality in a safe way.

Another aspect associated with the use of male condoms was cohabitation. The influence of living with both parents has also been observed in relation to other behaviors in adolescence, such as the onset of sexual life^3^ and seems to be linked to good communication between parents and their teenagers about health in general, but also to sexual health and contraception^13^.

In turn, studying at a private school – which in Brazil can be considered an indicator of better social and economic status – was associated with the use of dual protection in the last sexual intercourse. Despite this, the economic group variable did not show any influence on the use of methods among adolescent men, either male condoms or dual protection. This leads us to believe there is something in private schools that influences the use of contraceptive methods, and not just purchasing power. The school is understood as a unique setting for the inclusion of sexuality education as a cross-curricular activity, to advance in discussions about life and the experience of being a teenager, including the exercise of sexuality^23^. Private educational institutions seem to be more permeable to this type of approach and preparation of adolescents for the exercise of their sexual lives.

The relationship between alcohol consumption, sexual practices and use of contraceptive methods among adolescents is controversial. Studies show that alcohol can have a negative effect on male condom use among adolescents: in Felson et al.^24^, adolescent men who reported a higher frequency of alcohol use were 50% less likely to use male condoms, when compared to young men who did not report recurrent alcohol use. Likewise, for Brazilian adolescents from a city in the South of the country, the use of male condoms was statistically lower among adolescents who reported using alcohol compared to those who did not^25^. Adolescents under the influence of alcohol focus more on their own desire and less on the risks of pregnancy and STIs, reducing the possibility of using contraceptive methods and, thus, becoming sexually vulnerable^24^.

However, this study found that alcohol consumption in the previous 30 days was associated with male condom use, probably because of the way alcohol intake was measured. A meta-analysis on the effect of alcohol on casual sexual experiences among young people showed inconsistency in the associations; partly because alcohol use was assessed as consumption or frequency in the last month prior to the survey or in the moment prior to sexual intercourse^26^. The fact we do not have a way to verify the actual consumption of alcohol before the last intercourse is a limitation presented by this research. It could be that the use of alcohol or tobacco occurred after the investigated event; however, as the study design is cross-sectional, there is no way to establish any causal relationship.

The use of alcohol and tobacco, even in adolescence, tends to occur simultaneously, as they have similar determinants^27^. An assessment of this simultaneity of behaviors found a relationship with not using condoms, more frequent in younger, non-white boys who did not live with their parents^28^. In this study, there was no association with simultaneous consumption of alcohol and tobacco, but smoking alone had an inverse effect on the use of dual protection, a result similar to that found in Mexico, albeit among adolescent women^21^.

Results presented here can be seen as an expression of different logics that permeate sexual intercourse, a sanitary one and a contraceptive one. The HIV/AIDS epidemic introduced a new logic of protection in sexual relationships, an inescapable dimension for younger generations, especially due to the public prevention policies that different countries have adopted since the late 1980s. The issue of exercising sexuality in a safe and healthy manner as a right of men and women, including the youth population, also emerges with force on the national and international scene. In this context, public policies, especially in the field of education and health, started to be promoted so that the condom, traditionally invoked for its preventive power against “venereal diseases”, could be introduced in sexual relations in general. Successive campaigns and/or government actions have been underway since then, as well as the monitoring of levels of male condom use (mainly in the first and last sexual intercourse), which are measured in different types of studies. However, the emphasis on its specificity as a device for preventing STIs has always been the main focus of such actions, leaving aside its potential to perform the dual function of contraceptive and preventive resource.

Although our results increase knowledge about the use of contraceptive methods in adolescent men, one of the main limitations of this study is that it is not able to verify the consistency of male condom use, whether it occurred in homo-affective or hetero-affective relationships, and whether the use was with the objective of preventing STIs, pregnancy or both, i.e., if it was used as a contraceptive, a preventive resource, or both. Evidence indicates the use of male condoms is more related to prevention against STIs and, therefore, it occurs more frequently in the beginning of affective-sexual relationships or in unstable relationships^29^.

Furthermore, one does not know to what extent the report of the use of oral hormonal contraceptives, in the case of dual protection, was trustworthy or underestimated, since this is a method of female use, it would be subject to the report of use by the partner. It does not seem casual to us that the proportion of dual protection (combined use of a condom with another contraceptive method) is so low among the young participants in this study. The dichotomy between a sanitary logic and a contraceptive one maintains (and even reinforces) the concept that responsibilities for contraception are a female concern. The low proportion of dual protection in the last sexual intercourse may reflect this male lack of knowledge about a role that has historically been attributed to women.

Despite these limitations, the study has strengths. This is a robust sample of Brazilian adolescent students covering all regions of the country and refers to a pioneering analysis of the use of dual protection by adolescent men, a rarely studied group. Our data allow for comparison with national and international studies, as the use of contraceptive methods in the last sexual intercourse is the event frequently considered to measure current practices. The results add evidence to the national and middle-income countries literature on the use of dual protection and associated aspects among adolescent men.

Future studies will benefit and provide even more elements that clarify male behavior, if they consider from their starting point/in their premises, the crossing of different logics in sexual relations. We suggest that the questioning about the use of a method, whether in the first or last sexual intercourse, should be accompanied by questioning on the type of partnership existing at the time of the sexual act^30^, giving greater intelligibility to statements about double protection or other methods.

## CONCLUSION

The use of male condoms and dual protection among Brazilian adolescent men was quite diverse. We indicate the need to understand the results of this study from different logics that govern sexual relationships. One must deconstruct the classic dichotomy present in public policy formulations (and in some investigations) that the sphere of sexuality is of the domain/interest of men, while that of reproduction concerns almost exclusively women.

The national and international sociopolitical context, mainly a conservative shift, especially in the last decade, has generated apprehension in specialists dedicated to the field of studies on youth sexuality. In Brazil, this conservative turn has promoted a continuous suppression or dismantling of previously existing initiatives of sex education and STI/HIV/AIDS and pregnancy prevention programs in schools, which will result in fewer opportunities for safe and healthy exercise of sexuality.
