# Supplementary material for: Male condom and dual protection use by adolescent men in Brazil
Source: Rev Saude Publica. 2021 Nov 23;55:109. doi: 10.11606/s1518-8787.2021055003298 (PMC8621551; doi:10.11606/s1518-8787.2021055003298)
Supplement: Translation

# Translation: Uso de preservativo masculino e dupla proteção por homens adolescentes no Brasil

**Authors:** Ana Luiza Vilela Borges, Luciane Simões Duarte, Cristiane da Silva Cabral, Alejandra Andrea Roman Lay, Osmara Alves Viana, Elizabeth Fujimori

**Affiliations:** I São Paulo SP Brasil Universidade de São Paulo. Escola de Enfermagem. Departamento de Enfermagem em Saúde Coletiva. São Paulo, SP, Brasil; II São Paulo SP Brasil Secretaria de Estado da Saúde de São Paulo. Centro de Vigilância Epidemiológica. Divisão de Doenças Crônicas Não Transmissíveis. São Paulo, SP, Brasil; III São Paulo SP Brasil Universidade de São Paulo. Faculdade de Saúde Pública. Departamento Saúde, Ciclos de Vida e Sociedade. São Paulo, SP, Brasil; IV Arica Chile Universidad de Tarapacá. Facultad de Ciencias de la Salud. Arica, Chile

**Keywords:** Adolescente, Homens, Anticoncepção, Anticoncepcionais Orais, Preservativos, Saúde Sexual e Reprodutiva

## Abstract

**OBJETIVO:**

Analisar o uso de preservativo masculino e de dupla proteção por homens adolescentes brasileiros, bem como os aspectos associados.

**MÉTODOS:**

Utilizou-se banco de dados do Estudo de Riscos Cardiovasculares em Adolescentes (ERICA) para este estudo transversal nacional de base escolar. A amostra incluiu adolescentes de ambos os sexos, com idades entre 12 e 17 anos de idade, selecionados por meio de amostragem por conglomerados, em 2014 (n = 75.060). O presente estudo analisou informações dos homens adolescentes que relataram já ter tido relação sexual (n = 12.215), tendo como variáveis dependentes o uso de preservativo masculino e o uso de dupla proteção (uso simultâneo de preservativo masculino e contraceptivo hormonal oral) na última relação sexual. Os dados foram analisados por meio de regressão logística univariada e múltipla.

**RESULTADOS:**

A maior parte dos adolescentes usou preservativo masculino na última relação sexual, enquanto o uso de dupla proteção foi bastante baixo. O uso de preservativo masculino, referido por 71% (IC95% 68,7–73,1), associou-se positivamente à idade, a coabitar com ambos os pais e a ter usado álcool nos 30 dias anteriores. O uso de dupla proteção, referido por 3,6% (IC95% 2,8–4,5) associou-se positivamente à idade e a estudar em escola privada e negativamente ao uso de tabaco nos 30 dias anteriores.

**CONCLUSÕES:**

A larga diferença apresentada na proporção de uso de preservativo ou de dupla proteção na última relação sexual chama atenção para as distintas lógicas que presidem as relações sexuais juvenis. A baixa proporção aqui encontrada de uso da dupla proteção pode ser reflexo do desconhecimento masculino sobre uma função que tem sido historicamente atribuída às mulheres, que é a contracepção. Dessa forma, é necessário desconstruir a dicotomia de que a esfera da sexualidade é de domínio/interesse dos homens enquanto a da reprodução concerne apenas às mulheres.

## INTRODUÇÃO

As práticas contraceptivas entre adolescentes têm se mostrado bastante heterogêneas entre os países de alta, média e baixa renda, mas têm em comum o fato de se limitarem ao uso de métodos de curta duração, como o preservativo masculino e, em menor proporção, o contraceptivo hormonal oral^1–4^. O conhecimento sobre o comportamento contraceptivo na adolescência advém majoritariamente de estudos que consideram apenas mulheres^5^. Isso ocorre por diversas razões: em geral, inquéritos populacionais são realizados predominantemente com mulheres; as estatísticas vitais de nascimentos trazem apenas dados das mulheres que são mães, mas não dos pais; os métodos contraceptivos amplamente disponíveis são basicamente de uso feminino; as taxas de gestação na adolescência são estimadas considerando em seu denominador apenas mulheres na faixa etária adolescente. Em resumo, a principal razão de os estudos considerarem apenas as mulheres ao pesquisarem comportamento contraceptivo na adolescência é que as mulheres continuam sendo responsabilizadas pelo planejamento reprodutivo e o cuidado com os filhos^6,7^ em nossa sociedade. A diminuta presença dos homens no universo das pesquisas sobre contracepção reflete a invisibilidade masculina em políticas públicas e programas de saúde^7^.

O método contraceptivo mais utilizado por adolescentes homens é o preservativo masculino^1,3,4^. Além da prevenção de gravidez, o preservativo masculino é altamente recomendado para adolescentes visando à prevenção de infecções sexualmente transmissíveis (IST) e HIV/AIDS, tanto por ser de baixo custo como por ser amplamente disponível. Dados do estudo *Health Behavior in School-aged Children* (HBSC), que envolveu 42 países, principalmente europeus, mostraram que 65% dos adolescentes de 15 anos, de ambos os sexos, tinham usado o preservativo masculino na última relação sexual^8^. Resultado próximo foi registrado entre adolescentes norte-americanos (54%)^9^. No Brasil, os dados da Pesquisa Nacional de Saúde do Escolar (PeNSE), realizada com adolescentes de 13 a 17 anos, destacam patamares elevados do uso de preservativo na última relação sexual, apesar de significativa queda no intervalo de tempo entre dois momentos da pesquisa (era 76% em 2009 e caiu para 66% em 2015)^10^.

O recurso à dupla proteção (uso do preservativo masculino associado a outro método contraceptivo para prevenir a gravidez) é pouco conhecido entre homens adolescentes^3,11^. No Brasil, apesar de não haver informações específicas sobre a prática entre adolescentes homens, dados da PeNSE 2015 constataram prevalência de 29,7% de uso de dupla proteção entre adolescentes que haviam iniciado a vida sexual^10^.

Este artigo analisa o uso de preservativo masculino e de dupla proteção entre adolescentes homens brasileiros, bem como seus aspectos associados. Pretende-se contribuir para elucidação de lacunas na literatura sobre as práticas contraceptivas de homens, incluindo os adolescentes. Prover cuidados baseados em evidências aos homens adolescentes é importante para contemplar suas necessidades de saúde sexual e reprodutiva, incluindo a prevenção de IST, a contracepção e a paternidade^12^. Indubitavelmente, a prática de sexo seguro entre adolescentes permanece como um grande desafio para a área da saúde sexual e reprodutiva, frente às evidências de aumento nas taxas de HIV/Aids e IST^8,13^ e das altas taxas de fecundidade adolescente no Brasil e América Latina^14^.

## MÉTODOS

Este é um estudo transversal de base escolar nacional, que utilizou o banco de dados do Estudo dos Riscos Cardiovasculares em Adolescentes (ERICA). O ERICA entrevistou 75.060 adolescentes de ambos os sexos, que frequentavam escolas de cidades brasileiras com mais de 100 mil habitantes, a fim de avaliar a prevalência de condições de risco cardiovascular^15^.

Os adolescentes foram selecionados, em 2014, por meio de amostragem por conglomerados, considerando escola, turno, ano e turma de instituições públicas e privadas. A amostra incluiu adolescentes com idades entre 12 e 17 anos, do ensino fundamental (7º ao 9º ano) e do ensino médio (1º ao 3º ano). Os procedimentos de cálculo e plano amostral encontram-se publicados^16^. O presente estudo analisou informações referentes aos homens adolescentes que relataram já ter tido relação sexual (n = 12.215), o que representa 16,3% da amostra.

Dados sociodemográficos e comportamentais foram coletados por meio de *Personnal Digital Assistant* e o questionário foi autopreenchido pelos adolescentes nas salas de aula, sob supervisão da equipe^15^.

As variáveis dependentes foram o uso de preservativo masculino e o uso de dupla proteção na última relação sexual, dados obtidos por meio da questão “Da última vez que você teve relação sexual, você ou seu(sua) parceiro(a) utilizaram (pode marcar mais de uma opção): nunca tive relação sexual; camisinha; pílula anticoncepcional; pílula do dia seguinte; outro”. Dupla proteção foi entendida como uso simultâneo de preservativo masculino (camisinha) e contraceptivo hormonal oral (pílula anticoncepcional), ou seja, quando as duas opções foram assinaladas. Não se considerou a contracepção de emergência (pílula do dia seguinte) por não se tratar de método de uso regular.

As variáveis independentes reunindo as informações sobre as características sociodemográficas e comportamentais foram: idade (em anos), cor da pele (branca ou não branca, que incluiu preta, parda, amarela e indígena), residência (capital ou interior), região do Brasil (Norte, Nordeste, Sudeste, Sul ou Centro-Oeste), coabitação (nem com a mãe e nem pai, somente com mãe, somente com pai ou ambos), rede de escola (pública ou privada), trabalho remunerado (não ou sim), grupo econômico (baixo, médio ou alto) e uso de álcool/tabaco nos 30 dias anteriores (não, apenas tabaco, apenas álcool ou ambos). Os grupos econômicos foram construídos com base nos bens de consumo que os adolescentes possuíam em casa, de acordo com os procedimentos descritos para se calcular o *Wealth Index* nas pesquisas do tipo *Demographic and Health Survey*^17^, considerando os bens de consumo televisão, rádio, máquina de lavar, DVD, geladeira, freezer, motocicleta, carro e computador. Também foi considerada a contratação de empregada doméstica trabalhando no domicílio.

Os dados foram descritos por meio de proporções e intervalos de confiança de 95% (IC95%). Aspectos associados ao uso de preservativo masculino e de dupla proteção na última relação sexual foram, primeiramente, analisados por meio de regressão logística univariada e, posteriormente, múltipla, com entrada simultânea das variáveis no modelo. Uma vez que se trata de amostra complexa, os pesos amostrais foram considerados em todas as análises. Foi considerado nível de significância de 5%. Todas as análises foram conduzidas no Stata 15.0.

O ERICA foi aprovado pelos Comitês de Ética em Pesquisa do Instituto de Estudos em Saúde Coletiva da Universidade Federal do Rio de Janeiro (Processo n. 45/2008) e de cada um dos 26 estados e do Distrito Federal.

## RESULTADOS

Dentre os 12.215 homens adolescentes com idades entre 12 e 17 anos que relataram já ter tido relação sexual, a maior parte tinha entre 15 e 17 anos (70,5%), e autoclassificou a cor de sua pele como não branca (66,2%), frequentava escolas públicas (86,9%), residia no interior (56,2%) e não exercia qualquer trabalho remunerado (56,9%). Pouco mais da metade morava com ambos os pais (51,4%) e informou não ter usado tabaco nem álcool nos 30 dias anteriores à entrevista (52,4%). Quase metade residia na região Sudeste (49,4%) e estava incluído no grupo econômico médio (49,0%) (Tabela 1).

**Tabela 1:** Características sociodemográficas e comportamentais de adolescentes homens no Brasil. ERICA, Brasil, 2013–2014.

Características		Total (n = 12.215)		Uso de preservativo masculino (n = 8.783)		Uso de dupla proteção (n = 469)	
		%	IC95%	%	IC95%	%	IC95%
Idade (anos)							
	12	5,4	4,5–6,5	3,3	2,5–4,4	0,6	0,0–2,9
	13	9,6	8,4–10,9	8,1	6,8–9,6	1,7	0,1–4,3
	14	14,5	13,3–15,8	15,4	13,4–17,3	6,6	39,7–10,7
	15	22,3	20,7–23,9	22,5	20,7–24,5	25,1	16,5–36,3
	16	24,3	22,9–25,8	25,3	23,5–27,1	27,6	21,4–34,7
	17	23,9	22,6–25,2	25,4	23,6–27,2	38,4	31,3–46,1
Rede escolar							
	Pública	86,9	82,5–90,3	86,9	82,8–90,2	76,8	61,5–87,3
	Privada	13,1	9,6–17,5	13,1	9,8–17,1	23,2	12,7–38,5
Cor da pele							
	Branca	33,8	31,7–35,9	33,7	31,3–36,2	43,4	31,0–56,7
	Não Branca	66,2	64,1–68,3	66,3	63,8–68,7	56,6	43,3–69,0
Residência							
	Capital	43,8	41,5–46,1	43,3	407–45,8	41,8	31,3–53,2
	Interior	56,2	53,9–58,5	56,7	54,1–59,3	58,2	468–68,7
Região do Brasil							
	Norte	10,5	9,8–11,2	10,3	9,7–11,2	7,3	4,9–10,5
	Nordeste	21,9	20,0–23,9	21,0	19,2–22,9	9,9	6,1–15,7
	Sudeste	49,4	46,8–52,0	50,0	47,1–52,8	57,7	46,2–68,5
	Sul	10,3	9,2–11,5	10,6	9,1–12,3	15,6	10,3–22,9
	Centro-Oeste	7,9	7,0–8,9	8,1	6,9–9,4	9,5	5,9–14,8
Coabitação							
	Nem com mãe e nem com pai	7,7	6,3–9,3	7,2	6,1–8,4	4,3	2,6–6,9
	Somente com mãe	35,2	33,3–37,3	34,8	32,6–37,1	40,2	29,1–52,5
	Somente com pai	5,7	5,0–6,6	5,3	4,4–6,3	5,5	27,5–10,7
	Ambos	51,4	48,7–54,0	52,7	50,0–55,4	50,0	38,1–61,8
Exercia trabalho remunerado							
	Não	56,9	55,1–58,7	56,4	54,0–58,7	47,7	36,1–59,5
	Sim	43,1	41,3–44,9	43,6	41,3–46,0	52,3	40,4–63,9
Grupo econômico							
	Baixo	28,8	26,6–31,0	27,6	25,5–29,9	16,9	11,2–24,6
	Médio	49,0	46,8–51,2	49,4	47,0–51,8	56,5	47,5–65,2
	Alto	22,2	19,9–24,7	23,0	20,4–25,8	26,6	19,2–35,5
Uso de álcool/tabaco nos 30 dias anteriores							
	Não	52,4	49,7–55,0	51,1	48,4–53,9	42,1	34,1–50,7
	Apenas tabaco	2,7	2,2–3,3	2,7	2,1–3,3	0,7	0,3–1,4
	Apenas álcool	34,5	32,1–36,9	36,2	33,7–38,9	47,0	38,2–56,0
	Ambos	10,4	9,2–11,8	10,0	8,6–11,5	10,2	5,9–17,0

A maior parte dos adolescentes usou preservativo masculino na última relação sexual (71,7%; IC95% 68,7–73,1). O uso de dupla proteção foi baixo (3,6%; IC95% 2,8–4,5).

A associação de características dos homens adolescentes com o uso de preservativo masculino na última relação sexual é apresentada na Tabela 2. A idade dos adolescentes associou-se ao uso de preservativo masculino (ORaj = 1,23; IC95% 1,16–1,31). Adolescentes que moravam com ambos os pais tiveram mais chance de usar o preservativo masculino contrastando àqueles que não moravam com nenhum deles (ORaj = 1,61; IC95% 1,04–2,49), assim como aqueles que usaram álcool nos 30 dias anteriores, quando comparados aos que não usaram nem tabaco nem álcool (ORaj = 1,29; IC95% 1,00–1,65).

**Tabela 2:** Associação entre características sociodemográficas e comportamentais de adolescentes homens no Brasil e uso do preservativo masculino na última relação sexual. ERICA, Brasil, 2013–2014.

Características		Uso de preservativo masculino			
		Análise univariada		Análise múltipla	
		OR_bruto_	IC95%	OR_aj_	IC95%
Idade (anos)		1,21	1,14–1,29	1,23	1,16–1,31
Rede escolar					
	Pública	Ref.	-	Ref.	-
	Privada	0,99	0,75–1,31	0,85	0,60–1,20
Cor da pele					
	Branca	Ref.	-	Ref.	-
	Não branca	1,01	0,84–1,22	1,07	0,86–1,33
Região do Brasil					
	Norte	Ref.	-	Ref.	-
	Nordeste	0,90	0,71–1,14	0,83	0,65–1,06
	Sudeste	1,07	0,88–1,32	0,95	0,77–1,17
	Sul	1,14	0,84–1,55	1,02	0,72–1,44
	Centro-Oeste	1,12	0,86–1,46	1,01	0,83–1,37
Residência					
	Capital	Ref.	-	Ref.	-
	Interior	1,07	0,88–1,30	1,00	0,81–1,22
Coabitação					
	Nem com mãe e nem com pai	Ref.	-	Ref.	-
	Somente com mãe	1,18	0,77–1,80	1,41	0,91–2,19
	Somete com pai	0,94	0,62–1,44	0,98	0,63–1,55
	Ambos	1,35	0,85–2,02	1,61	1,04–2,49
Exercia trabalho remunerado					
	Não	Ref.	-	Ref.	-
	Sim	1,08	0,88–1,32	1,00	0,80–1,23
Grupo econômico					
	Baixo	Ref.	-	Ref.	-
	Médio	1,17	0,98–1,41	1,13	0,89–1,43
	Alto	1,29	1,01–1,67	1,32	0,97–1,77
Uso de tabaco/álcool nos 30 dias anteriores					
	Não	Ref.	-	Ref.	-
	Apenas tabaco	1,01	0,66–1,53	1,02	0,64–1,63
	Apenas álcool	1,30	1,03–1,64	1,29	1,00–1,65^a^
	Ambos	0,93	0,71–1,21	0,94	0,71–1,26

OR: *odds ratio*; ORaj: *odds ratio* ajustado; Ref.: referência.

a p = 0,049

A Tabela 3 apresenta os fatores associados ao uso de dupla proteção na última relação sexual entre homens adolescentes. Os resultados mostraram que a idade também se associou ao uso de dupla proteção, assim como estudar em escola privada. O uso de tabaco/álcool nos 30 dias anteriores foi inversamente associado ao uso da dupla proteção (ORaj = 0,27; IC95% 0,10–0,69). Embora as variáveis sobre região de residência, coabitação e grupo econômico constassem como associadas ao uso de dupla proteção na análise univariada, elas perderam a significância no modelo múltiplo.

**Tabela 3:** Associação entre características sociodemográficas e comportamentais de adolescentes homens no Brasil e uso de dupla proteção na última relação sexual. ERICA, Brasil, 2013–2014.

Características		Uso de dupla proteção			
		Análise univariada		Análise múltipla	
		OR_bruto_	IC95%	OR_aj_	IC95%
Idade (anos)		1,50	1,31–1,71	1,44	1,25–1,67
Rede escolar					
	Pública	Ref.	-	Ref.	-
	Privada	2,07	1,18–3,63	1,72	1,04–2,82
Cor da pele					
	Branca	Ref.	-	Ref.	-
	Não branca	0,56	0,37–1,11	0,94	0,56–1,61
Região do Brasil					
	Norte	Ref.	-	Ref.	-
	Nordeste	0,65	0,39–1,10	0,62	0,36–1,07
	Sudeste	1,74	1,07–2,84	1,29	0,80–2,10
	Sul	2,29	1,44–3,63	1,65	0,97–2,81
	Centro-Oeste	1,79	1,01–3,15	1,50	0,84–2,67
Residência					
	Capital	Ref.	-	Ref.	-
	Interior	1,09	0,70–1,68	1,23	0,71–1,78
Coabitação					
	Nem com mãe nem com pai	Ref.	-	Ref.	-
	Somente com mãe	2,09	1,06–4,11	1,49	0,94–6,64
	Somete com pai	1,75	0,76–4,02	1,95	0,75–5,06
	Ambos	1,77	0,93–3,63	1,83	0,73–4,56
Exercia trabalho remunerado					
	Não	Ref.	-	Ref.	-
	Sim	1,46	0,89–2,40	1,27	0,77–2,10
Grupo econômico					
	Baixo	Ref.	-	Ref.	-
	Médio	2,02	1,20–3,39	1,53	0,86–2,70
	Alto	2,09	1,19–3,68	1,47	0,80–2,70
Uso de tabaco/álcool nos 30 dias anteriores					
	Não	Ref.	-	Ref.	-
	Apenas tabaco	0,29	0,13–0,66	0,27	0,10–0,69
	Apenas álcool	1,73	1,17–2,57	1,31	0,84–2,03
	Ambos	1,21	0,65–2,28	0,97	0,48–1,98

OR: *odds ratio*; ORaj: *odds ratio* ajustado; Ref.: referência.

## DISCUSSÃO

Os resultados do presente estudo, que analisou dados do ERICA, contribuem para o avanço do conhecimento sobre o uso de preservativo masculino e da dupla proteção entre homens adolescentes. Observou-se que o uso desses métodos aumenta com a idade e sofre influência da coabitação com os pais, do uso de álcool e/ou tabaco e da rede escolar em que o adolescente estuda.

Observando os dados obtidos sobre o uso do preservativo masculino, os resultados mostraram-se mais elevados que os verificados na PeNSE 2015^10^, que foi de 66%, e no *Youth Risk Behavior Survey* de 2017, que avaliou estudantes de 14 a 17 anos de idade nos Estados Unidos, que foi 61%^18^. Por outro lado, o uso foi menor do que observado em países europeus, que obtiveram resultados próximos aos 80%^19^. Do total de adolescentes homens selecionados nesta pesquisa, 71,7% usaram preservativo masculino em sua última relação sexual.

Contudo, o uso de dupla proteção na última relação sexual foi baixo, quando comparado a outros estudos. Em escolas públicas de Cuiabá, no Mato Grosso, por exemplo, 9% dos adolescentes homens do ensino médio referiram combinação de métodos contraceptivos^20^. Segundo a *National Survey of Family Growth*, essa proporção chegou a 19% entre os homens adolescentes norte-americanos^3^, porém dados do *Youth Risk Behavior Survey* mostraram que somente 9% usavam métodos duplos de proteção, proporção que tem se mantido constante desde 2013^9^. Diversos estudos analisam o uso de dupla proteção, considerando o uso de outros métodos além do preservativo masculino, como o preservativo feminino, dispositivo intrauterino, injetáveis e implantes, ao passo que nosso estudo considerou apenas o preservativo masculino e o contraceptivo hormonal oral, o que torna a comparação dos resultados delicada. De toda forma, o uso desses outros métodos não considerados aqui entre adolescentes tem sido pouco frequente no país^10^.

A idade foi o único aspecto que se associou tanto ao uso do preservativo masculino quanto de dupla proteção, confirmando que adolescentes mais velhos tendem a usar mais os métodos contraceptivos, o que já foi observado anteriormente em outras pesquisas^2,3,13^. Isso também evidencia que os adolescentes mais jovens podem ser os mais vulneráveis às consequências do não uso de métodos contraceptivos, como gestação não intencional, IST e HIV/Aids^3,21^. Um aspecto que pode ter contribuído para esses resultados seriam as barreiras na obtenção dos métodos contraceptivos, seja por dificuldade de acessar os serviços de saúde que os ofertam, seja por terem menos recursos financeiros para obtê-los em farmácias, além de dúvidas sobre como obter e usar os métodos contraceptivos^21^.

Considerando que o diálogo entre os parceiros sobre as consequências de uma relação sexual desprotegida, bem como explicitar o desejo de usar um método, são pré-requisitos essenciais para aumentar o uso do preservativo masculino entre homens adolescentes^22^, porém os mais jovens podem ter maior dificuldade e inexperiência para lidar com essas questões. Por essa razão, é importante reconhecer que ações de educação para sexualidade devem ser implementadas ainda no início da adolescência, antes mesmo do início da vida sexual, com acesso à informação e insumos para que homens adolescentes possam vivenciar sua sexualidade de forma segura.

Outro aspecto associado ao uso do preservativo masculino foi a coabitação. A influência de morar com ambos os pais já foi observada também em relação a outros comportamentos na adolescência, como o início da vida sexual^3^ e parece estar atrelada à boa comunicação dos pais com seus filhos adolescentes sobre saúde em geral, mas também à saúde sexual e contracepção^13^.

Por sua vez, estudar em escola privada – o que no Brasil pode ser considerado indicador de melhor inserção social e econômica – mostrou associação com o uso da dupla proteção na última relação sexual. Apesar disso, a variável grupo econômico não mostrou qualquer influência sobre o uso de métodos entre os homens adolescentes, seja preservativo masculino, seja dupla proteção. Isso nos leva a crer que haja algo nas escolas privadas que influencie o uso de métodos contraceptivos e não apenas o poder aquisitivo. Compreende-se a escola como cenário singular para inclusão da educação para a sexualidade como atividade curricular transversal, de forma a avançar nas discussões sobre a vida e a experiência de ser adolescente, incluindo o exercício da sexualidade^23^. As instituições privadas de ensino parecem ser mais permeáveis a esse tipo de abordagem e de preparação dos adolescentes para o exercício da vida sexual.

A relação entre consumo de álcool, práticas sexuais e uso de métodos contraceptivos entre os adolescentes é controversa. Estudos apontam que o álcool pode ter efeito negativo sobre o uso de preservativo masculino entre adolescentes: em Felson et al.^24^, homens adolescentes que relataram maior frequência de uso de álcool tinham 50% menos chance de usar preservativo masculino, quando eram comparados aos jovens que não indicaram o uso recorrente de álcool. Da mesma forma, para adolescentes brasileiros de uma cidade do Sul do País, o uso de preservativo masculino foi estatisticamente menor entre os adolescentes que relataram uso de álcool em comparação com os que não usaram álcool^25^. Adolescentes sob o efeito do álcool focam mais o próprio desejo e menos os riscos de gravidez e IST, diminuindo a possibilidade de usar métodos contraceptivos e tornando-se sexualmente vulneráveis^24^.

Entretanto, neste estudo, verificou-se que o consumo de álcool nos 30 dias anteriores foi associado ao uso do preservativo masculino, provavelmente, por causa da forma como a ingestão de álcool foi mensurada. Uma meta-análise sobre o efeito do álcool em experiências sexuais casuais entre jovens apontou inconsistência nas associações, em parte, porque se avaliou o uso de álcool como consumo ou frequência no último mês anterior à pesquisa ou no momento anterior à relação sexual^26^. Não termos como averiguar o real consumo de álcool antes da última relação é uma limitação apresentada por esta pesquisa. Pode ser que o uso de álcool ou tabaco tenha ocorrido depois do evento investigado; porém, como o delineamento do estudo é transversal, não há como estabelecer qualquer relação de causalidade.

O uso de álcool e tabaco, inclusive na adolescência, tende a ocorrer de forma simultânea, pois possuem determinantes semelhantes^27^. Avaliação dessa simultaneidade de comportamentos constatou uma relação com o não uso de preservativo, mais frequente em meninos mais novos, não brancos e que não moravam com os pais^28^. Neste estudo, não se observou associação com a simultaneidade de consumo de álcool e tabaco, mas o tabagismo isolado teve efeito inverso no uso da dupla proteção, resultado similar ao encontrado no México, mas entre adolescentes mulheres^21^.

Os resultados aqui apresentados podem ser compreendidos como expressão de distintas lógicas que atravessam os intercursos sexuais, uma lógica sanitária e outra contraceptiva. A epidemia do HIV/Aids introduziu uma nova lógica de proteção nas relações sexuais, dimensão inescapável para as gerações mais jovens, sobretudo em função das políticas públicas de prevenção que os diversos países adotaram, desde o final da década de 1980. Eclode também com força no cenário nacional e internacional o tema do exercício da sexualidade de forma segura e saudável enquanto um direito de homens e mulheres, incluindo a população juvenil. Nesse contexto, políticas públicas, sobretudo no campo da educação e da saúde, passaram a ser fomentadas para que o preservativo, tradicionalmente invocado por seu poder preventivo de “doenças venéreas”, fosse introduzido nas relações sexuais de modo geral. Sucessivas campanhas e/ou ações governamentais são postas em curso desde então, bem como o acompanhamento dos níveis de uso do preservativo masculino (principalmente na primeira e na última relação sexual) são mensurados em diferentes tipos de estudos. Contudo, a ênfase na sua especificidade como um dispositivo de prevenção às IST sempre foi o enfoque principal de tais ações, ficando à margem sua potencialidade em desempenhar a dupla função de recurso anticonceptivo e preventivo.

Embora nossos resultados ampliem o conhecimento que se tem sobre o uso de métodos contraceptivos em homens adolescentes, uma das principais limitações deste estudo é não poder averiguar a consistência do uso do preservativo masculino, se ocorreu em relacionamentos homoafetivos ou heteroafetivos, e se o uso foi com objetivo de se prevenir IST, gravidez ou ambos, ou seja, se foi usado como recurso anticonceptivo, preventivo ou ambos. Evidências apontam que o uso do preservativo masculino está mais relacionado à prevenção contra IST e, por isso, ocorre mais frequentemente no início de relacionamentos afetivo-sexuais ou em relacionamentos não estáveis^29^.

Além disso, não se sabe o quanto o relato do uso do contraceptivo hormonal oral, no caso da dupla proteção, foi fidedigno ou subestimado, já que, por se tratar de método de uso feminino, estaria sujeito ao relato de uso pela parceira. Não nos parece casual que a proporção de dupla proteção (uso combinado do preservativo com outro método contraceptivo) seja tão baixa entre os jovens participantes do presente estudo. A dicotomia entre uma lógica sanitária e uma contraceptiva mantém (e até mesmo, reforça) a concepção de que os encargos da contracepção são femininos. A baixa proporção de declaração de uso da dupla proteção na última relação sexual pode ser reflexo desse desconhecimento masculino sobre uma função que tem sido historicamente atribuída às mulheres.

Apesar dessas limitações, o estudo possui pontos fortes. Trata-se de uma amostra robusta de adolescentes brasileiros contemplando todas as regiões do país e refere-se a uma análise pioneira de uso de dupla proteção por homens adolescentes, grupo pouco estudado. Nossos dados permitem comparabilidade com estudos nacionais e internacionais, pois o uso de método contraceptivo na última relação sexual é o evento frequentemente considerado para se mensurar as práticas correntes. Os resultados adicionam evidências à literatura nacional e à dos países de média renda sobre o uso de dupla proteção e aspectos associados entre homens adolescentes.

Estudos futuros se beneficiarão e aportarão ainda mais elementos elucidativos do comportamento masculino, caso considerem, desde seu ponto de partida/em suas premissas, o atravessamento de lógicas distintas nas relações sexuais. Sugere-se ainda que a interrogação sobre uso de método, seja na primeira ou na última relação sexual, deva ser acompanhada do questionamento sobre o tipo de parceria existente no momento do ato sexual^30^, dando maior inteligibilidade às declarações sobre dupla proteção ou de outros métodos.

## CONCLUSÃO

O uso de preservativo masculino e dupla proteção entre homens adolescentes brasileiros mostrou-se bastante diverso. Indicamos a necessidade de compreender os resultados do presente estudo a partir de lógicas distintas que presidem as relações sexuais. É necessário desconstruir a clássica dicotomia presente nas formulações de políticas públicas (e em algumas investigações) de que a esfera da sexualidade é de domínio/interesse dos homens, enquanto a da reprodução concerne quase exclusivamente às mulheres.

O contexto sociopolítico nacional e internacional, em especial uma guinada conservadora, especialmente na última década, gera apreensão em especialistas dedicados ao campo de estudos sobre sexualidade juvenil. No Brasil, esse giro conservador tem promovido uma contínua supressão ou desmonte de iniciativas outrora vigentes de educação em sexualidade e de programas de prevenção de IST/HIV/AIDS e de gestação nas escolas, o que implicará em menos oportunidades para o exercício seguro e saudável da sexualidade.
